# Epidemiology of *Mycoplasma genitalium* in British men and women aged 16–44 years: evidence from the third National Survey of Sexual Attitudes and Lifestyles (Natsal-3)

**DOI:** 10.1093/ije/dyv194

**Published:** 2015-11-03

**Authors:** Pam Sonnenberg, Catherine A Ison, Soazig Clifton, Nigel Field, Clare Tanton, Kate Soldan, Simon Beddows, Sarah Alexander, Rumena Khanom, Pamela Saunders, Andrew J Copas, Kaye Wellings, Catherine H Mercer, Anne M Johnson

**Affiliations:** ^1^Research Department of Infection and Population Health, University College London, London, UK; ^2^Sexually Transmitted Bacteria Reference Unit, Public Health England, London, UK; ^3^NatCen Social Research, London, UK; ^4^Centre for Infectious Disease Surveillance and Control (CIDSC); ^5^Virus Reference Department, Public Health England, London, UK; ^6^Department of Social and Environmental Research, London School of Hygiene and Tropical Medicine, London, UK

**Keywords:** *Mycoplasma genitalium*, STI, epidemiology, population, sexual behaviour

## Abstract

**Background:** There are currently no large general population epidemiological studies of *Mycoplasma genitalium* (MG), which include prevalence, risk factors, symptoms and co-infection in men and women across a broad age range.

**Methods:** In 2010-–12, we conducted the third National Survey of Sexual Attitudes and Lifestyles (Natsal-3), a probability sample survey in Britain. Urine from 4507 sexually-experienced participants, aged 16–44 years, was tested for MG.

**Results:** MG prevalence was 1.2% [95% confidence interval (CI): 0.7–1.8%] in men and 1.3% (0.9–1.9%) in women. There were no positive MG tests in men aged 16–19, and prevalence peaked at 2.1% (1.2–3.7%) in men aged 25–34 years. In women, prevalence was highest in 16–19 year olds, at 2.4% (1.2–4.8%), and decreased with age. Men of Black ethnicity were more likely to test positive for MG [adjusted odds ratio (AOR) 12.1; 95% CI: 3.7–39.4). For both men and women, MG was strongly associated with reporting sexual risk behaviours (increasing number of total and new partners, and unsafe sex, in the past year). Women with MG were more likely to report post-coital bleeding (AOR 5.8; 95%CI 1.4–23.3). However, the majority of men (94.4%), and over half of women (56.2%) with MG did not report any sexually transmitted infection (STI) symptoms. Men with MG were more likely to report previously diagnosed gonorrhoea, syphilis or non-specific urethritis, and women previous trichomoniasis.

**Conclusions:** This study strengthens evidence that MG is an STI. MG was identified in over 1% of the population, including in men with high-risk behaviours in older age groups that are often not included in STI prevention measures.

Key Messages

- This study strengthens evidence that MG is an STI: there were strong associations with risky sexual behaviours, with behavioural risk factors similar to those in other known STIs, and no infections were detected in those reporting no previous sexual experience.
- Given the uncertainty on the natural history and clinical implications of infection, especially in women, here we report that although asymptomatic infection was common, we found a strong association with post-coital bleeding in women. Thus in addition to MG being an STI, it can be an STD.
- MG was identified in over 1% of the population aged 16–44, and among men was most prevalent in 25–34-year-olds, who would not be included in STI prevention measures aimed at young people.

## Introduction

There is growing evidence that *Mycoplasma genitalium* (MG) is a cause of non-specific urethritis (NSU) in men, and a putative cause of genital tract disease in women, although its natural history and clinical sequelae remain unclear.^1–3^ Our knowledge of this organism, which was first isolated in the 1980s, has been enhanced by the development of DNA amplification assays which can now be used in clinical settings and large-scale epidemiological studies. Guidelines for MG testing, treatment and control procedures, both on an individual clinical level and a population level, are needed. These will evolve as the evidence base accumulates and commercial testing for MG becomes more widely available.^4^^,^^5^

There are currently no data on the epidemiology of MG in any general population sample that extends the age range beyond 27 years and includes both men and women (Table 1).^6–10^ Much of our current understanding of the transmission dynamics of MG, including associations with sexual behaviour, is derived from studies conducted in selected populations, such as HIV-positive women,^11^ and is limited to young people.^6–10^ These include settings such as sexual health clinics, primary care, chlamydia screening programmes and further education colleges.

**Table 1.** Community-based studies of Mycoplasma genitalium

Setting	Study participants	Age	Sample size	Specimen	Prevalence	Risk factors	Reference
**MEN**							
US	National Longitudinal Study of Adolescent Health (Add Health)	18–27	1218	Urine	1.1% (0.5–2.4)	(Men and women combined): ever lived with a partner, Black ethnicity, ever had vaginal intercourse, ≥ 2 lifetime partners, ≥ 1 partner in the past year, condom use at last vaginal intercourse	Manhart *et al.*^7^
Denmark	Chlamydia Screening Programme	21–23	731	Urine	1.1% (0.3–1.9)	Younger age at first sex, > 6 partners in the past year, > 3 partners in the past 6 months	Andersen *et al.*^6^
**WOMEN**							
US	National Longitudinal Study of Adolescent Health (Add Health)	18–27	1714	Urine	0.8% (0.4–1.6)	See above	Manhart *et al.*^7^
London	Chlamydia Screening Programme	15–24	1424	Urine	1.0% (0.6–1.7)	(For all specimen types combined): Black ethnicity, > 1 partner in the past year	Svenstrup *et al.*^9^
London	Chlamydia Screening Programme	15–24	1017	Cervical/ vaginal swab	4.1% (3.0–5.5)	See above	Svenstrup *et al.*^9^
Denmark	Chlamydia Screening Programme	21–23	921	Vulvo-vaginal swab	2.3% (1.3–3.2)	> 10 lifetime partners, ≥ 2 partners in the past year, shorter duration of steady relationship, partner with symptoms	Andersen *et al.*^6^
Australia	General practice	15–25	1116	Vulvo-vaginal swab	1.6% (0.7–2.6)	Indigenous status, ≥ 2 partners in the past year, ≥ 3 partners in the past year without condoms	Walker *et al.*^10^
London	Students (POPI trial)	15–27	2378	Vulvo-vaginal swab	3.3% (2.6–4.1)	≥ 2 partners in the past year, ≤ 18 years, Black ethnicity, smoking	Oakeshott *et al.*^8^

Using data from Britain’s third National Survey of Sexual Attitudes and Lifestyles (Natsal-3), we have previously described the epidemiology of *Chlamydia trachomatis* (CT), *Neisseria gonorrhoeae* (GC), high-risk human papillomavirus (HR-HPV) and HIV in the British population and reported associations with sexual behaviour.^12^ In this paper, we aimed to describe the epidemiology of MG in British men and women aged 16–44 and explore its characteristics as a potential STI (through combining prevalence and behavioural data; comparison with other known STIs; occurrence of co-infection; and reported symptoms), to inform STI control strategies.

## Methods

### Participants and procedure

Natsal-3 was a stratified probability sample survey of 15 162 men and women aged 16–74 years in Britain (England, Scotland and Wales), interviewed in 2010–12. The overall response rate was 57.7% of all eligible addresses, with 65.8% of potential respondents at eligible addresses where contact was made agreeing to take part in the survey (the cooperation rate). Participants were interviewed using computer-assisted face-to-face and self-completion (CASI) questionnaires, which included questions on participants’ sexual lifestyles, history of STIs and current STI symptoms. Full details of Natsal-3 methods, including power calculations, are described elsewhere.^13^^,^^14^

### Urine collection

Following the interview, a sample of participants (all 16–17-year-olds and a sample of 18–44-year-olds who reported at least one sexual partner, ever) were invited to provide a urine specimen. Sample size calculations assumed an estimated prevalence of 1.5% for MG, which would give at least 80% power to estimate the prevalence within 0.6% in each gender and univariate associations. Of the 8047 respondents aged 16–44 years who reported at least one sexual partner, ever, 4828 (60.0%) agreed to provide a sample to be tested for a range of infections.^12^ Urine test results for MG were available on 4507 samples. We also obtained urine samples from 205 of the 406 respondents aged 16–17 years who had reported never having had vaginal, anal or oral sex, and we have an MG result on 189 of these participants. Urine was collected using the FirstBurst device, which collects the first 4–5 ml of voided urine, yielding a higher CT organism load than the regular urine cup,^15^ which is likely to also increase detection of MG.

### Laboratory methods

Samples were posted to Public Health England (PHE) for testing. Details of urine sample preparation, testing for CT, GC, HPV and HIV, and quality assurance, are available elsewhere.^13^^,^^14^ For MG, urine specimens were tested using an in-house real-time polymerase chain reaction (PCR) assay which targets the *Mycoplasma genitalium* adhesin protein (MgPa) gene,^16^ with positive or equivocal results confirmed using the Aptima *Mycoplasma genitalium* test (RUO, Hologic Inc., San Diego, USA).^17^

### Statistical analysis

All analyses were done in Stata v13, accounting for stratification, clustering and weighting of the sample.^13^^,^^14^ In order to minimize non-participation bias, the Natsal-3 data were weighted in two stages. The first stage corrects for participants’ unequal probabilities of selection for inclusion in the sample, with weights applied which were inversely proportional to the selection probabilities for the numbers of households and adults within the eligible age range at each selected address. The second stage adjusts for differential non-response by comparing the age, gender and regional profile of participants with 2011 Census data. The final weighted sample closely matches the distribution of demographic characteristics in the 2011 Census. In order to reduce bias in the urine results, we included an additional ‘urine weight’, which is the product of a weight that corrects for differential urine sample response after already weighting for different selection probabilities.^13^^,^^14^ The urine non-response weight is the inverse of the predicted probability of urine response from a logistic regression model, which is based on forwards stepwise model selection conducted separately among men and women, and with age included a priori. Differences in the provision of a urine sample were small. In general, those who were younger, who reported same-sex experience and higher-risk behaviours, such as more unprotected partners, were more likely to provide a urine sample.

We present weighted prevalence estimates of MG in men and women, by age group, with 95% confidence intervals (CIs), in participants aged 16–44 years who reported at least one sexual partner, ever. We examined the relationships between MG and demographic and behavioural variables using logistic regression and present crude and adjusted odds ratios (ORs, AORs) and compared these with risk factors for other STIs (CT and HR-HPV), that we have previously reported.^12^ Multivariable analyses adjusted for two demographic variables [age and area-level deprivation (IMD)]^18^ and one behavioural factor (number of sexual partners in the past year). Analyses of number of new partners in the past year and unsafe sex (defined as sex with two or more partners in the past year and never used condoms in the past year), were only adjusted for age and IMD, due to collinearity with total number of partners in the past year. We examined the association between MG and reported previous STI diagnoses, current co-infection, and STI symptoms over the past month, and present AORs. To provide further evidence about whether MG is sexually transmitted, we report whether MG was detected in urine in the 16–17 year olds who had not had sex, and in those reporting at least one partner, ever, but only having oral sex.

### Ethics

We obtained ethical approval from Oxfordshire Research Ethics Committee A (No. 09/H0604/27). Participants gave written informed consent to anonymized testing without the return of results, the ethical rationale for which has been previously described.^19^ To avoid potential deductive disclosure to others in the household, urine was requested from all participants aged 16–17 years irrespective of sexual experience. All participants were provided with information on where to obtain free diagnostic STI/HIV testing and sexual health advice.

## Results

### Prevalence

We detected MG in urine from 24 sexually experienced men and 48 sexually experienced women, giving a weighted prevalence of 1.2% (95% CI: 0.7–1.8%) in men and 1.3% (0.9–1.9%) in women aged 16–44 (Table 2) [combined weighted prevalence in men and women aged 16–44 was 1.2% (0.9–1.7)]. There were no positive MG tests in men aged 16–19, and prevalence peaked at 2.1% in men aged 25–34 years. In contrast, in women prevalence was highest in 16–19-year-olds, at 2.4%, and decreased with age. The prevalences in those aged 16–24 years were 0.4% (0.1–1.1%) in men and 1.7% (1.1–2.6%) in women [combined weighted prevalence in men and women aged 16–24 was 1.0% (0.7–1.5)]. Over 90% of MG in men [90.6% (75.8–96.8%)] and two-thirds of MG in women [66.5% (50.3–79.5%)] was in those aged 25–44 years.

**Table 2.** Socio-demographic risk factors for *Mycoplasma genitalium* in urine in participants aged 16–44, by gender

	Men							Women							*Denominator(unweighted, weighted)*	
	%	(95% CI)	OR	(95% CI)	AOR^a^	(95% CI)	*P*-value	%	(95% CI)	OR	(95% CI)	AOR^a^	(95% CI)	*P* -value	*Men*	*Women*
All aged 16—44 years	1.2%	(0.7–1.8)						1.3%	(0.9–1.9)						*1875, 2252*	*2632, 2248*
Age at interview (years)							0.025							0.694		
16–19	0.0%	–	–	–	–	–		2.4%	(1.2–4.8)	2.43	(0.74–7.93)	1.58	(0.51–4.87)		*342, 233*	*391, 211*
20–24	0.6%	(0.2–1.7)	0.78	(0.17–3.53)	0.35	(0.06–1.96)		1.3%	(0.7–2.3)	1.30	(0.44–3.88)	0.94	(0.33–2.71)		*493, 388*	*591, 379*
25–34	2.1%	(1.2–3.7)	2.68	(0.77–9.26)	1.78	(0.46–6.89)		1.4%	(0.8–2.3)	1.41	(0.49–4.06)	1.30	(0.46–3.71)		*691, 803*	*1132, 799*
35–44	0.8%	(0.3–2.4)	1.00	–	1.00	–		1.0%	(0.4–2.5)	1.00	–	1.00	–		*349, 828*	*518, 859*
Ethnicity							<0.001							0.408		
White	0.7%	(0.4–1.3)	1.00	–	1.00	–		1.4%	(1.0–2.0)	1.00	–	1.00	–		*1684, 1921*	*2368, 1965*
Asian	1.0%	(0.1–7.2)	1.47	(0.18–11.71)	1.20	(0.15–9.62)		0.0%	–	–	–	–	–		*66, 156*	*87, 121*
Black	11.2%	(4.8–24.1)	17.57	(5.88–52.48)	12.05	(3.68–39.44)		2.1%	(0.5–8.4)	1.50	(0.34–6.68)	1.29	(0.31–5.44)		*56, 86*	*78, 78*
Mixed/other	1.3%	(0.2–8.8)	1.80	(0.23–14.27)	1.43	(0.17–11.67)		0.4%	(0.1–3.2)	0.31	(0.04–2.32)	0.27	(0.04–2.00)		*69, 88*	*99, 85*
Region							0.796							0.732		
London	1.1%	(0.3–4.5)	1.00	–	1.00	–		1.1%	(0.4–3.2)	1.00	–	1.00	–		*180, 334*	*266, 340*
Rest of Britain	1.2%	(0.7–1.9)	1.04	(0.23–4.66)	1.21	(0.29–5.08)		1.3%	(0.9–2.0)	1.22	(0.39–3.79)	1.24	(0.36–4.23)		*1695, 1918*	*2366, 1909*
IMD (quintiles)^b^							0.053							0.304		
1–2 (least deprived)	0.5%	(0.2–1.1)	1.00	–	1.00	–		1.4%	(0.7–2.8)	1.00	–	1.00	–		*677, 835*	*894, 799*
3	0.9%	(0.3–2.8)	1.94	(0.48–7.80)	2.01	(0.49–8.26)		0.7%	(0.3–1.6)	0.46	(0.16–1.35)	0.47	(0.16–1.40)		*368, 464*	*514, 449*
4–5 (most deprived)	1.9%	(1.0–3.4)	3.97	(1.41–11.18)	3.66	(1.27–10.47)		1.5%	(1.0–2.3)	1.04	(0.46–2.35)	0.98	(0.43–2.22)		*830, 952*	*1224, 1001*
Academic qualifications^c^							0.429							0.616		
No academic qualifications	2.9%	(1.0–8.2)	1.00	–	1.00	–		2.0%	(0.7–5.6)	1.00	–	1.00	–		*171, 221*	*222, 199*
Academic qualifications typically gained at age 16^d^	1.0%	(0.5–2.2)	0.34	(0.09–1.34)	0.43	(0.12–1.55)		1.1%	(0.7–2.0)	0.56	(0.17–1.84)	0.54	(0.15–1.97)		*630, 775*	*895, 761*
Studying for / attained further academic qualifications	1.0%	(0.5–2.0)	0.36	(0.10–1.30)	0.57	(0.18–1.86)		1.2%	(0.7–2.1)	0.59	(0.18–1.94)	0.52	(0.13–2.10)		*969, 1157*	*1382, 1192*

Denominator is participants aged 16–44 who reported at least one partner, with an MG test result.

^a^Adjusted for age, IMD quintile and number of partners in the past year, except for age group (adjusted for IMD and partners only).

^b^IMD, Index of Multiple Deprivation, a multidimensional measure of area (neighbourhood)-level deprivation based on the participant's postcode. We adjusted IMD scores for England, Scotland and Wales before they were combined and assigned to quintiles, with use of a method by Payne and Abel.^17^

^c^Participants aged ≥ 17 years.

^d^English General Certificate of Secondary Education (GCSE) or equivalent.

### Risk factors

Table 2 shows demographic risk factors for MG. Men of Black ethnicity (AOR 12.05; 95% CI 3.68–39.44) and those living in the most deprived areas (AOR 3.66; 1.27–10.47) were more likely to test positive for MG. When additionally adjusting for ethnicity, the association between MG positivity and area-level deprivation in men reduced to an AOR of 2.83, although this was no longer statistically significant (AOR adjusted for age, number of partners in the past year and ethnicity: 2.83;0.87–9.21). We did not find any significant associations with socio-demographic factors in women. For both men and women, MG was strongly associated with reporting a range of sexual risk behaviours (increasing number of total and new partners, and unsafe sex, in the past year) (Table 3), although the magnitudes of the AORs were greater in men than in women. Women, but not men, reporting ever having same-sex experience were more likely to test MG-positive (OR 2.80; 1.09–7.22), and this association remained after adjusting for age, IMD and number of partners, albeit not statistically significantly (AOR 2.10; 0.72–6.07).

**Table 3.** Behavioural risk factors for *Mycoplasma genitalium* in urine in participants aged 16–44, by gender

	Men							Women							*Denominator (unweighted, weighted)*	
	%	(95% CI)	OR	(95% CI)	AOR^a^	(95% CI)	*P*-value	%	(95% CI)	OR	(95% CI)	AOR^a^	(95% CI)	*P*-value	*Men*	*Women*
All aged 16–44 years	1.2%	(0.7–1.8)						1.3%	(0.9–1.9)						*1875, 2252*	*2632, 2248*
Age at first sex,^b^ years							0.114							0.002		
16+	0.7%	(0.3–1.4)	1.00	–	1.00	–		0.7%	(0.4–1.3)	1.00	–	1.00	–		*1153, 1510*	*1710, 1623*
< 16	2.1%	(1.1–3.8)	3.22	(1.19–8.72)	2.23	(0.82–6.03)		2.9%	(1.9–4.6)	4.10	(1.97–8.52)	3.66	(1.61–8.32)		*688, 706*	*888, 598*
Number of sexual partners,^c^ past year							0.001							0.005		
0–1	0.6%	(0.3–1.1)	1.00	–	1.00	–		1.0%	(0.6–1.7)	1.00	–	1.00	–		*1268, 1721*	*1974, 1850*
2–3	2.2%	(0.9–5.1)	3.97	(1.26–12.47)	4.96	(1.41–17.49)		2.7%	(1.5–4.8)	2.77	(1.26–6.11)	2.57	(1.24–5.35)		*376, 340*	*424, 257*
4+	5.2%	(2.3–11.3)	9.80	(3.21–29.89)	11.69	(3.14–43.54)		3.1%	(1.6–5.9)	3.19	(1.37–7.46)	2.90	(1.32–6.35)		*222, 180*	*214, 125*
Number of new sexual partners,^c^ past year							0.005							0.061		
0	0.6%	(0.3–1.2)	1.00	–	1.00	–		1.1%	(0.6–1.8)	1.00	–	1.00	–		*1053, 1537*	*1770, 1703*
1+	2.4%	(1.3–4.4)	3.94	(1.58–9.83)	5.02	(1.62–15.58)		2.1%	(1.4–3.2)	2.02	(1.02–4.01)	1.82	(0.97–3.41)		*812, 703*	*842, 528*
Unsafe sex, past year^d^							0.001							0.006		
No	0.8%	(0.5–1.4)	1	–	1.00	–		1.2%	(0.8–1.8)	1	–	1.00	–		*1724, 2110*	*2382, 2094*
Yes	5.9%	(2.2–14.5)	7.77	(2.52–24.01)	7.29	(2.19–24.19)		3.6%	(1.9–6.8)	3.17	(1.44–6.98)	2.95	(1.37–6.36)		*143, 132*	*236, 142*
Ever had same-sex experience involving genital contact							0.143							0.172		
No	1.2%	(0.7–1.9)	1.00	–	1.00	–		1.1%	(0.8–1.7)	1.00	–	1.00	–		*1738, 2131*	*2337, 2055*
Yes	0.4%	(0.1–3.1)	0.36	(0.05–2.70)	0.21	(0.03–1.69)		3.1%	(1.3–7.1)	2.80	(1.09–7.22)	2.10	(0.72–6.07)		*137, 121*	*295, 194*

Denominator is participants aged 16–44 who reported at least one partner, with an MG test result.

^a^Adjusted for age, IMD quintile and number of partners in the past year, except for number of new partners in the past year and unsafe sex (adjusted for age and IMD only due to collinearity).

^b^Age at first heterosexual intercourse or first same-sex experience involving genital contact.

^c^Includes both opposite-sex and same-sex partners.

^d^Had sex with two or more partners in the past year and didn't use a condom at all in the past year.

**Figure 1.**
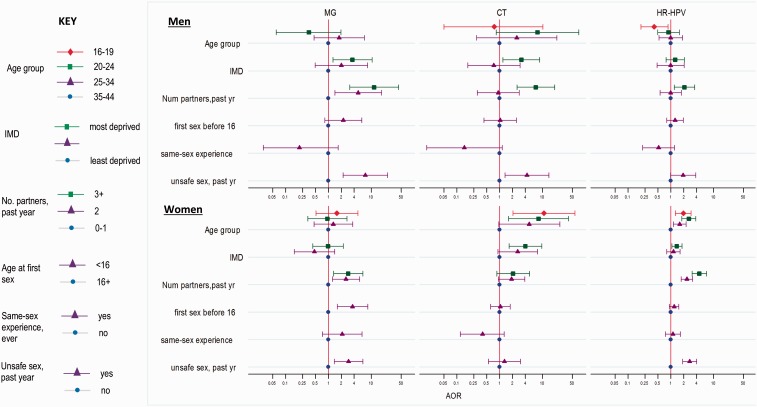
Risk factors for *Mycoplasma* genitalium (MG), *Chlamydia trachomatis* (CT) and high-risk HPV (HR-HPV), by gender; yr, year; num, number. AOR, adjusted odds ratio, adjusted for age, IMD and number of partners in the past year, except ‘unsafe sex’ which is adjusted for age and IMD only. Unsafe sex = 2+ partners in past year and not used a condom at all in past year.

Figure 1 compares AORs for a number of risk factors for MG, CT and HR-HPV by gender. Apart from age, and in women ever having same-sex experience, the direction and magnitude of risks were broadly similar for MG, CT and HR-HPV.

### Previous STI diagnoses and current co-infection

Men with MG were more likely to report having been diagnosed with gonorrhoea, syphilis and/or NSU/NGU (non-gonococcal urethritis) in the past 5 years than those MG-negative, with women being more likely to report a diagnosis of trichomoniasis, although numbers are small (Table 4). Men and women who were MG-positive were more likely to have HR-HPV, and any HPV, detected in urine than those MG-negative. Only two respondents (one man and one woman) had both MG and CT detected in urine. One man had MG and GC co-infection.

**Table 4.** Associations between MG in urine, self-reported STI diagnoses in the past 5 years and CT and HR-HPV in urine, by gender

	MG-negative		MG-positive		OR	95% CI	*P*-value
Men							
Self-reported previous diagnoses: % (95% CI)							
Diagnosed with an STI in past 5 years^a^	5.5%	[4.6%,6.7%]	10.4%	[3.5%,26.8%]	1.97	(0.61–6.38)	0.257
Chlamydia	3.6%	[2.8%,4.6%]	10.4%	[3.5%,26.8%]	3.09	(0.95–10.05)	0.060
Gonorrhoea	0.3%	[0.1%,0.6%]	3.6%	[0.8%,13.9%]	12.88	(2.43–68.19)	0.003
Genital warts	1.3%	[0.8%,1.9%]	3.6%	[0.8%,13.9%]	2.88	(0.61–13.49)	0.179
Syphilis	0.2%	[0.1%,0.6%]	2.0%	[0.3%,13.4%]	10.98	(1.06–113.33)	0.044
Trichomoniasis	0.0%	–	0.0%	–	–	–	–
Herpes	0.4%	[0.2%,0.8%]	0.0%	–	–	–	–
NSU/NGU	0.8%	[0.5%,1.2%]	3.6%	[0.8%,13.9%]	4.88	(1.01–23.52)	0.048
Urine prevalence: % (95% CI)							
*Chlamydia trachomatis* in urine	1.1%	[0.8%,1.6%]	3.8%	[0.5%,22.9%]	3.69	(0.47–28.85)	0.182
HR-HPV in urine	8.1%	[6.5%,10.1%]	35.0%	[16.2%,60.0%]	6.10	(2.13–17.45)	<0.001
HPV (any type) in urine)	16.0%	[13.9%,18.3%]	40.6%	[20.6%,64.3%]	3.60	(1.35–9.60)	0.006
Women							
Self-reported previous diagnoses: % (95% CI)							
Diagnosed with an STI in past 5 years^a^	6.0%	[5.1%,7.1%]	8.5%	[3.8%,18.0%]	1.45	(0.61–3.43)	0.397
Chlamydia	4.1%	[3.5%,4.9%]	3.2%	[1.0%,9.9%]	0.76	(0.23–2.56)	0.660
Gonorrhoea	0.4%	[0.2%,0.8%]	0.0%	–	–	–	–
Genital warts	1.4%	[1.0%,1.9%]	4.2%	[1.3%,13.0%]	3.09	(0.88–10.83)	0.077
Syphilis	<0.1%	[0.0%,0.1%]	0.0%	–	–	–	–
Trichomoniasis	0.1%	[0.0%,0.2%]	1.1%	[0.2%,7.7%]	14.70	(1.53–140.88)	0.020
Herpes	0.9%	[0.6%,1.4%]	1.3%	[0.2%,9.1%]	1.53	(0.21–11.00)	0.672
Urine prevalence: % (95% CI)							
*Chlamydia trachomatis* in urine	1.5%	[1.1%,2.0%]	1.2%	[0.2%,8.2%]	0.82	(0.11–6.27)	0.848
HR-HPV in urine	15.6%	[14.0%,17.2%]	41.0%	[25.4%,58.6%]	3.77	(1.82–7.78)	<0.001
HPV (any type) in urine)	30.2%	[28.1%,32.5%]	68.3%	[47.8%,83.5%]	4.96	(2.11–11.68)	<0.001
Denominator (unweighted, weighted)							
Men	(1833, 2202)		(24, 26)				
Women	(2568, 2201)		(48, 29)				

Denominator is participants aged 16–44 who reported at least one partner, with an MG test result. MG-negative is the reference category.

^a^Diagnosed with chlamydia, gonorrhoea, genital warts, syphilis, trichomoniasis, herpes in the past 5 years.

### Symptoms

The majority of men [94.4% (79.4–98.7%)] and over half [56.2% (37.5–73.3%)] of women with MG did not report any STI symptoms in the past month. In men there were no associations between reported STI symptoms and MG positivity. Women who tested positive for MG were more likely to report STI symptoms in the past month than those who tested negative (Table 5) (43.8% vs 25.7%; OR 2.26; 1.05–4.84) and this association remained after adjusting for CT, GC or HR-HPV co-infection (AOR 2.19; 1.00–4.78; *P* = 0.05). When looking at specific symptoms, women with MG were significantly more likely to report having experienced bleeding after sex (OR 6.15; 1.64–23.09; AOR adjusted for age, current CT, GC or HR-HPV 5.78 (1.43–23.34); *P* = 0.014).

**Table 5.** Reported STI symptoms in the past month, among those with and without MGin urine, and gender

	MG-negative		MG-positive		OR		*P*-value	AOR		*P*-value
Men										
Any STI symptom: % (95% CI)	7.3%	[6.1%,8.9%]	5.6%	[1.3%,20.6%]	0.75	(0.17–3.35)	0.706	0.68	(0.15–3.09)	0.619
Pain, burning or stinging passing urine	2.3%	[1.7%,3.2%]	2.6%	[0.4%,16.7%]	1.13	(0.15–8.69)	0.909	0.80	(0.10–6.23)	0.813
More frequent urination	2.5%	[1.8%,3.6%]	0.0%	–	–	–		–	–	–
Genital warts/lumps	0.8%	[0.4%,1.5%]	0.0%	–	–	–		–	–	–
Genital ulcers/sores	0.2%	[0.1%,0.6%]	0.0%	–	–	–		–	–	–
Penile discharge	0.3%	[0.2%,0.7%]	0.0%	–	–	–		–	–	–
Painful testicles	2.8%	[2.1%,3.7%]	3.0%	[0.4%,19.0%]	1.09	(0.14–8.48)	0.936	1.43	(0.18–11.10)	0.731
Women										
Any STI symptom: % (95% CI)	25.7%	[23.7%,27.7%]	43.8%	[26.7%,62.5%]	2.26	(1.05–4.84)	0.037	2.15	(0.92–5.00)	0.077
Pain, burning or stinging passing urine	7.8%	[6.6%,9.0%]	7.8%	[1.1%,38.2%]	1.00	(0.14–7.40)	0.999	0.87	(0.10–7.41)	0.900
More frequent urination	5.2%	[4.3%,6.3%]	8.6%	[1.5%,36.3%]	1.72	(0.28–10.49)	0.558	1.49	(0.22–10.15)	0.682
Genital warts/lumps	0.8%	[0.5%,1.4%]	0.0%	–	–	–		–	–	–
Genital ulcers/sores	0.7%	[0.4%,1.3%]	1.8%	[0.3%,12.0%]	2.73	(0.34–22.18)	0.347	2.13	(0.27–16.76)	0.470
Abnormal vaginal discharge	4.0%	[3.3%,4.9%]	0.9%	[0.1%,6.3%]	0.22	(0.03–1.61)	0.135	0.18	(0.03–1.35)	0.096
Odorous vaginal discharge	4.0%	[3.2%,5.0%]	5.0%	[1.5%,15.7%]	1.27	(0.35–4.54)	0.718	1.11	(0.32–3.78)	0.871
Vaginal pain during sex	6.7%	[5.6%,8.1%]	9.1%	[2.7%,26.7%]	1.39	(0.38–5.12)	0.618	1.29	(0.34–4.83)	0.706
Bleeding between periods	4.5%	[3.7%,5.5%]	7.1%	[1.6%,25.9%]	1.62	(0.35–7.51)	0.541	1.73	(0.36–8.39)	0.493
Bleeding after sex	2.7%	[2.0%,3.5%]	14.4%	[4.4%,38.2%]	6.15	(1.64–23.09)	0.007	5.78	(1.43–23.34)	0.014
Lower abdominal / pelvic pain	6.3%	[5.2%,7.7%]	10.8%	[3.7%,27.6%]	1.80	(0.57–5.72)	0.318	1.65	(0.53–5.17)	0.390
Denominator (unweighted, weighted)										
Men	(1849, 2222)		(24, 26)							
Women	(2578, 2215)		(48, 29)							

Denominator is those aged 16–44 who reported at least one sexual partner, ever. MG-negative is the reference category.

AOR, adjusted odds ratio, adjusted for chlamydia, gonorrhoea, HR-HPV infection, and age.

### MG in participants who were not sexually-experienced or only reported oral sex

MG test results were available from 89 men and 100 women aged 16–17 years who reported not having had sex, all of which were negative. There were also no positive MG tests in sexually experienced participants aged 16–44 years who only reported having had oral sex (*n* = 20 men and 22 women).

## Discussion

This population-based study, which includes men and women across a broad age range, found an overall prevalence of MG in urine from sexually-experienced people of 1.2% in men and 1.3% in women, and associations with a range of sexual behaviours and with other STIs.

There are different opinions on whether MG is a putative or confirmed STI. *M. genitalium* has been shown to be sexually transmissible, as evidenced by partner studies which demonstrate concurrent infection with concordant strain types.^20^^,^^21^ The cumulative epidemiological evidence in the literature is increasingly supporting the view that it is an STI (Table 1). This study adds to that evidence and gives further insights into the transmission dynamics of this infection. We detected no MG in those who had not had sex, or who only reported oral sex, although we may not expect to detect positives since: we found no MG in sexually experienced 16–19-year-old men; numbers are small; and the overall prevalence is low. Similarly, the study in US adolescents reported much higher prevalence in those reporting ever having had vaginal intercourse compared with those who had not.^7^We do show that, on a population level, MG was associated with a range of behavioural risk factors, as others have found,^6^^,^^7^^,^^9^^,^^10^ including increasing number of partners and unsafe sex. For example, prevalence was much higher in those reporting more partners (5.2% in men and 3.1% in women reporting four or more partners in the past year). The high ORs suggest that MG may be associated with same-sex experience in women, but not men, although confidence intervals are wide. All of the women reporting same-sex experience reported also having had sex with men, however one had only had sex with women in the past 5 years. The one man with MG reported having sex exclusively with men for the past 5 years. The association between same-sex sexual practices and MG may be better studied in specific populations, such as clinic attendees, than in national surveys.

The prevalence estimates and risk factors identified in this study are consistent with those obtained from the other community-based studies, such as those reported from urine from young people in the US National Longitudinal Study of Adolescent Health^7^ and from a recent London-based study in women aged 15–24 undergoing population-based chlamydia screening^9^ (Table 1). Nevertheless, there are potential biases in cross-sectional surveys with complex sampling designs, and other methodological challenges in sexual behaviour survey research,^22^ which will influence behavioural and STI prevalence estimates. It is difficult to assess survey non-response bias or how it impacts on prevalence estimates, given that little is known about non-responders. A recent international review found that surveys with lower response rates tended to produce higher chlamydia prevalence estimates. However, there was no evidence that this was the case for studies such as Natsal, with nationally representative samples.^23^ After weighting the Natsal-3 data to the British population Census data on age, sex and region, we found the profile of our sample was broadly comparable to the population in terms of ethnicity, marital status and self-reported general health.^13^^,^^14^ In contrast to non-participation bias to the survey, bias from non-provision of biological samples as a part of larger behavioural surveys can be better assessed, because demographic and behavioural data are known regarding those who take part in the survey but do not provide samples. We used these data to minimize the potential bias from sample non-provision by generating additional urine weighting.

The absolute prevalences in women are likely to be an underestimate, as MG is less likely to be detected from urine than vaginal swabs,^9^^,^^24^ although in our study this may be partially rectified by using the FirstBurst collection system. Natsal-3, in common with other studies using urine,^6^^,^^7^ is able to determine relative prevalences and associations with risk factors, other STIs and symptoms. The study had sufficient power to estimate prevalence and univariate associations. We present multivariable analyses that are only adjusted for a limited number of demographic and behavioural variables, and recognize that these AORs, given their wide confidence intervals, need to be interpreted with caution.

However, we found that the overall prevalence masks the heterogeneityof risk in the population, according to demographic and behavioural factors. For example, in men prevalence of MG was highest at 2.1% in those aged 25–34, whereas in women it peaked at 2.4% in those aged 16–19 years. Comparison of the epidemiology of MG in men and women, and with other STIs, may provide insights into the broader epidemiology, drivers of transmission and appropriate interventions. Nevertheless, most studies to date have either been limited to women^8–10^ or have combined analyses for men and women.^7^ We found that the magnitudes of associations between MG and socio-demographic and behavioural variables were greater in men than women. In women MG was more evenly distributed, and others have reported (based on small numbers of women) that MG may circulate in sexual networks different from those of CT or GC.^10^^,^^25^ It has been suggested that MG in women may require broad-based strategies for prevention since it seems that high rates of partner change are not necessarily required to sustain transmission.^25^ In contrast, we identified MG in men with high-risk behaviours, who had a history of GC, syphilis and NSU, which suggests that a control strategy that includes targeting this core group would be needed. The different findings in men and women may be due to chance, given the low prevalence. They may also reflect differences in age mixing, as men on average have partners younger than themselves.^26^ It is also possible, in a population-based survey, to have missed the women with highest risk behaviours or another ‘bridging population’.

The results from this study also generate a number of hypotheses. In addition to social and behavioural factors such as age and ethnic mixing, the transmission dynamics and natural history may differ in men and women. This may reflect biological and host factors that result in differences in the risk of infection following exposure, duration of infectiousness, development of symptoms, likelihood of persistence, rates of clearance and the role of reinfection. Furthermore, comorbidity with and treatment for other STIs may influence both the clinical course of MG infection and the development of drug resistance. The detailed sexual behaviour data collected in Natsal-3, combined with these MG results, provide parameter estimates for mathematical models that explore the transmission dynamics of this infection.

We found a strong association with detection of MG and Black ethnicity in men (OR 17.6), which remained after adjusting for age, IMD and number of partners in the past year (AOR 12.1). In addition to social and behavioural factors that result in higher risk in this group, there may be ethnic differences in innate immunity^27^ or in the vaginal microbiome,^28^ which may influence the risk of acquisition or persistence of infection. This finding is consistent with those reported from other studies^7^^,^^9^^,^^25^ and may be one explanation for the higher reported prevalence and incidence of MG in the POPI trial in London students with a high proportion of participants of Black ethnicity.^8^ Furthermore, surveillance data for England show high rates of STI diagnoses in persons of Black ethnicity.^29^ As ethnicity is a predictor of *Trichomonas vaginalis* infection,^25^ this may partly explain why we observed an association between MG and a history of trichomoniasis in women. A better understanding of sexual mixing patterns would inform whether intensified STI prevention and treatment interventions, including enabling access to services, may be warranted in those of Black ethnicity.

We found that the risk factors for MG were broadly similar to those for two known STIs, CT and HR-HPV. Men and women who were MG-positive were more likely to have HR-HPV and any HPV detected in urine than those who were MG-negative, which may be due to HR-HPV being a proxy measure for riskier sexual behaviour.^12^ We found little MG/CT co-infection and the prevalence of CT was not significantly different in those with and without MG, as reported elsewhere.^6^^,^^7^^,^^9^ However, the power to detect a significant association between MG and CT was limited since the prevalences are low. The magnitudes of the ORs suggest that men with MG are more likely to have current or to report previous CT, but not women, although again confidence intervals are wide and the association was not significant.

This study provides insights for the testing and control of MG infection, which need to be considered as part of the emerging evidence base. Other study designs are better placed to look at natural history of infection with *M. genitalium* and its sequelae. However, in order to ascertain whether in addition to it being an STI it is an STD (i.e. causes disease), our findings on symptoms are informative. Women who tested positive for MG were more likely to report symptoms than those who tested negative; however, over half (56.2%) of women with MG did not report any STI symptoms. Of note is that women with MG were more likely (OR 6.2) to report post-coital bleeding, which may result from cervical friability or cervicitis,^30^ and this strong association remained after adjusting for age and co-infection. We did not, however, find that women with MG were more likely to report other symptoms that are usually associated with pelvic inflammatory disease, such as pelvic pain, abnormal vaginal discharge or dyspareunia. Over 90% of men with MG did not report any STI symptoms and men with MG were not more likely to report current symptoms than those who tested negative, although they were more likely to report a previous diagnosis of NSU (OR 4.9). These findings suggest that only testing men who are currently symptomatic would miss the vast majority of infections. An understanding of the clinical implications of infection, together with information on resistance patterns to guide antibiotic choice, will inform recommendations on how to manage infection in symptomatic patients or in those who present to health services.^4^^,^^5^

There is the possibility that with rapidly-evolving diagnostic technologies, which include testing for multiple organisms on a single specimen (e.g. triple CT/GC/MG assays), testing for MG becomes the default both in clinical care and population screening. However, testing in settings with low prevalence are more likely to have low positive predictive values with false-positive results.^31^The overall prevalence of MG in those aged 16–44 years is similar to the prevalence of chlamydia in this population (1.1% in men and 1.5% in women).^12^ However, there are differences in the age- and sex-specific prevalences of these two infections which have important implications for control strategies. The prevalences of MG and CT in those aged 16–24 years were 0.4% and 2.3% in men and 1.7% and 3.1% in women, respectively. Our data show that a hypothetical MG screening programme targeting people aged 16–24 years (such as the National Chlamydia Screening Programme) would only identify 1 in 10 men and 1 in 3 women with MG, as the majority of MG-positive men and women were in the 25–44 year age group. General STI prevention measures that promote a reduction in risk behaviour (e.g. increased condom use) are likely to impact on all STIs, including MG. Further research in clinical and community-based settings, including those that explore sexual networks, the molecular epidemiology of MG, age mixing and other partnership factors across a range of STIs, will elucidate the transmission dynamics of MG. This will inform the development of any further control strategies, if these are deemed appropriate and cost-effective.

## Funding

Natsal-3 is a collaboration between University College London, the London School of Hygiene and Tropical Medicine, NatCen Social Research, Public Health England (PHE; formerly the Health Protection Agency) and the University of Manchester. The study was supported by grants from the Medical Research Council [G0701757] and the Wellcome Trust [084840], with contributions from the Economic and Social Research Council and Department of Health. N.F. is supported by an NIHR Academic Clinical Lectureship.
